# The Possible as a Field of Inquiry

**DOI:** 10.5964/ejop.v14i3.1725

**Published:** 2018-08-31

**Authors:** Vlad Petre Glăveanu

**Affiliations:** a Department of Psychology, Webster University Geneva, Geneva, Switzerland

**Keywords:** possible, imagination, creativity, counterfactuals, anticipation, sociocultural psychology

## Abstract

In this editorial I introduce the possible as an emerging field of inquiry in psychology and related disciplines. Over the past decades, significant advances have been made in connected areas – counterfactual thinking, anticipation, prospection, imagination and creativity, etc. – and several calls have been formulated in the social sciences to study human beings and societies as systems that are open to possibility and to the future. However, engaging with the possible, in the sense of both becoming aware of it and actively exploring it, represents a subject in need of further theoretical elaboration. In this paper, I review several existing approaches to the possible before briefly outlining a new, sociocultural account. While the former are focused on cognitive processes and uphold the old dichotomy between the possible and the actual or real, the latter grows out of a social ontology grounded in notions of difference, positions, perspectives, reflexivity, and dialogue. In the end, I argue that a better understanding of the possible can help us cultivate it in both mind and society.

The notion of the possible has a long history in philosophy and psychology, even if the type of phenomena it designates has often been named differently. What is undisputable, at minimum, is the fact that humans live not only in the here and now, but are capable of enriching and expanding their experience of the world by remembering the past, imagining the future, considering alternatives, anticipating problems, and continuously engaging in ‘as if’, ‘what if’ and ‘if only’ thinking processes. The common denominator between these experiences is the capacity to go beyond the actual or the real and to explore the possible. Which doesn’t mean that the possible is completely outside of reality or opposed to it. Indeed, both our sense of possibility and its exploration are grounded in what is already actualized and, most of all, transforms our experience of the actual. To live with(in) the possible is not a pathological state; on the contrary, it helps us adapt and grow within ever-changing physical and social environments. The key questions for those who recognize the possible as a field of inquiry is how to best define, study, and cultivate it for self and others.

Various answers to these interrogations have been offered through the centuries. In Antiquity, it was considered that philosophy has its origins in wonder (see Plato’s *Theaetetus*; Bostock, 1988), a state in which we start examining reality beyond what is given or what is visible. During the Renaissance, Pico della Mirandola, in his *Oration on the dignity of man* (della Mirandola, 1496/2012), argued that human beings are defined by their openness to the possible and not predetermined by nature. A similar line of argument has been developed in the 20^th^ century by existentialists such as Sartre (1996), who famously articulated the consequence of this view: that we are all condemned to be free. But what exactly does it mean to be ‘open’ and ‘free’, and how do these two relate to the possible? And what happens in experiences of the possible such as wonder? These definitional and process-based questions are of concern today across the social sciences, especially in recent years. In sociology, Tavory and Eliasoph (2013) stressed the fact that “any interaction includes a relationship to a future” (p. 909). In anthropology, Appadurai (2013), called for a more sustained study of imagination, anticipation, and aspiration. In psychology, Gergen (2015) thought research should generate possible futures – “not to illuminate what is, but to create what is to become” (p. 294).

The possible is thus rapidly emerging as an inter-disciplinary field of study with contributions from the social sciences and humanities, as well as from the natural sciences and the arts. Basically, all the disciplines that have an interest in anticipation, imagination, wonder and creativity, among others, can contribute unique perspectives to this old topic. Psychology is in a privileged position in this regard, as it has traditionally studied all these phenomena, including from different – sometimes radically different – perspectives. In this editorial, I will start by briefly reviewing theories and research on the possible in four key areas – possible worlds, possible selves, possible pasts, and possible future – before returning to the difficult questions of theory and its implications. Through the paper, it will become clear that there are at least two main approaches to the possible in psychology and connected disciplines. One focuses on *cognitive* processes and is concerned first and foremost with the dichotomy between the possible and the actual or the real (and, as a consequence, with how well cognitive processes can anticipate what can be and contribute to it). The other one, the *sociocultural* position, considers the possible as a relational phenomenon embedded within self–other, person–world relations. The emphasis here falls on differences of perspective and the way in which they create the necessary space for novelty to emerge. Towards the end, I will outline my own sociocultural framework for the study of the possible, not a definitive or comprehensive theory but one that could hopefully contribute to what is becoming today one of the most exciting fields of interdisciplinary research.

## Exploring Possible Worlds

One of the most obvious forms of engaging with the possible has to do with imagining the world differently than what it is or, in other words, imagining possible worlds. These possible words might differ in small ways from the actual one (for instance, we might wonder what would happen if we didn’t wake up on time for an important exam) or they can be fully imaginary, even impossible (like a fantastic universe). Importantly, there are *already* multiple worlds we experience, sometimes at once, on a daily basis – we effectively construct reality, at least in part, especially the reality of the societies we live in.

One of the best known philosophical arguments about possible worlds was formulated by Leibniz in early eighteenth century, when he famously claimed that our existing world is the best one God could have created (Leibniz, 1710/1760). In making this claim, he was trying to resolve the contradiction between claiming that God is omnipotent, omniscient, and omnibenevolent, and the presence of evil in God’s creation. While this view was satirically rejected by some and considered by many to reflect Leibniz’s optimism, it did contribute to another long-standing debate in philosophy regarding the ontological status (i.e., the reality) of possible worlds. Do they have an ‘objective’ existence (see Lewis, 1979) or do they always depend on the mind that is imagining them (see Rescher, 1979)? While the former could never be demonstrated, given the definition of possible worlds as not actual or actualized, the second position risks reinforcing the kind of solipsism that makes possibility an exclusive product of the mind. In the end, these ontological debates remain trapped within the strict separation (and the opposition) between the possible and the actual. This way of framing the possible might be good for definitional purposes, but it is counterproductive for understanding its role in the development of mind and society. A sharp distinction between the two invites questions regarding how close or how far the possible is from the actual, and even whether exploring it might actually disconnect us from reality.

There is a second dichotomy that is at play here, that between the ‘inner’ worlds constructed by the mind and the single, ‘outside’ world the person inhabits, together with others. In essence, following also Rescher’s (1979) argument, possible worlds are imagined ones. This doesn’t mean that they are completely disconnected from or inconsequential for the ‘real’ world. On the contrary, our imagination uses what the shared reality offers while transcending it by accessing the past, the future, the general, and the possible (Zittoun & Gillespie, 2016). Such imaginations, although seemingly subjective, build upon an intersubjective space of cultural resources (e.g., language and technology, among others), and often end up transforming ‘objective’ reality. In this sense, the psychic ontological status of possible worlds needs to be expanded in order to account for the material, the social, and the political, all of them crucial for the origin and the expression of possible worlds.

This is nowhere more obvious than in the case of imagining possible societies. In his well-known book, *The imaginary institution of society*, Castoriadis (1987) points us to the basic fact that our social organization, from its mundane interactions to its laws and institutions, is based on a series of myths explaining the world, how it came about, and what the place of man might be within it. These vast imaginaries transform through history, from traditional societies to communist and capitalist ones, but they also deeply communicate with each other (for instance, both communism and capitalism share the imaginary of the industrial revolution and the ideal of rationality). Ideology as a form of world-making is, indeed, essential for a deeper analysis of social and political life. It also comes to show very clearly the power of possible worlds in relation to the actual. Even when material conditions are similar, the way they are perceived and interpreted leads individuals and entire communities to different conclusions, different beliefs, and different courses of action. Ultimately, without grasping the imaginary institution of society, we would not be able to understand it as it is or why and how it transforms. This last point is important when it comes to social change and, in particular, to political art and creative activism (see Boros, 2012; Harrebye, 2016). In these contexts, it is not only different groups of people, animated by different goals, that seek to influence each other and shape society; what takes centre stage are their different views of what society is and, most of all, what it *could be*. Once more, possible worlds emerge from, respond to, but also get to drive, the constitution of ‘real’ ones.

## Exploring Possible Selves

The idea that human selves are possible selves is not new. As mentioned above, Pico della Mirandola (1496/2012) argued centuries ago that human beings don’t have a predetermined, fixed nature. On the contrary, they are beings open to the possible. A broader argument can be made that all living beings share, to different degrees, this kind of openness (and, in fact, any open system does). However, not every being is endowed with a self in the same way humans are. The human self emerges within a field of social relations and cultural resources, a field of actions and interactions that afford particular forms of agency and ways of relating to others, to one’s past and to the future. According to Bruner (1996, p. 36):

“What characterizes human selfhood is the construction of a conceptual system that organizes, as it were, a ‘record’ of agentive encounters with the world, a record that is related to the past (that is, ‘autobiographical memory’, so-called) but that is also extrapolated into the future – self with history and with possibility. It is a ‘possible self’ that regulates aspiration, confidence, optimism, and their opposites”.

The notion of possible selves has been used more than three decades ago by Markus and Nurius (1986) who defined it as conceptions of one’s self in future states. This conceptualization calls our attention to the important regulatory function of possible selves through the contribution they make to the person’s overall self-concept. It is not only the case that we consider who we are in the present, but do so at all times in view of the future and, importantly, of the multiple futures we can participate in. This latter precision is significant as it brings forth the issue of *agency*. As Erikson (2007) specifies, possible selves necessarily include an experience of being an agent in a future situation.

These clarifications are all useful, and yet the question arises of whether possible selves are only (envisioned) future ones. Going back to Bruner’s (1996) quote, we need to observe the role played by autobiographical memory and, more generally, the ways in which memory and imagination collaborate in expanding the self, at once, towards past and future. It is also important to recognize that future or imagined selves can become actualized and this process is, at least potentially, creative. Such dimensions are well captured by Zittoun and de Saint-Laurent’s (2015) concept of *life-creativity*. This notion points to the fact that selves are constructed and reconstructed, throughout the life-course, within imaginative explorations of the past, the future, and the possible. Considering the developmental dynamic of both the person and her context is of key significance here. Possible selves are not merely mental schemas or representations. They are forged within material, social and symbolic interactions and effectively transform these interactions. In this sense, possible selves have very real, tangible consequences for the development of the person and those around her.

Interestingly, possible selves are often enacted beyond the realm of imagination. They are embodied in play and role-playing episodes, in activities online and in the virtual world. Adopting different roles, including those of other or of different people, is part and parcel of daily, inter-personal interactions (see also Goffman, 1959), but it also finds notable, collective forms of expression during anime, sci-fi, or game conventions for instance. Most of all, online environments offer new opportunities for the development of virtual selves (Evans, 2012). The choice and use of avatars on social media and other interactive platforms connects to one’s image of oneself, both actual and possible. Leaving aside ongoing debates about the positive and negative effects of online participation, including for the user’s identity, self-concept and self-esteem, it is increasingly important to understand virtual selves, avatars and social media platforms within the context of possible selves and possible lives. While not always leading to an enhanced imagination or creativity (although the possibilities for this are ever-present, see Literat & Glăveanu, 2018), the use of technology – from using basic tools to the most sophisticated computers of today – has always accompanied human imagination, driven by a desire to expand the self’s immediate possibilities (Gillespie, Corti, Evans, & Heasman, 2017).

In the end, as Heidegger (1962) noted through his use of the term *Dasein*, the person doesn’t just live but does so through developing projects and being open to the future. Openness to others and to the world are fundamental dimensions of what it means to exist as a human self. Human beings exist, at all times, between the ‘here and now’ of their concrete life situation and the then and there of creativity, wonder, and imagination. In this manner, the self is, at once, *both* actual and possible. The opposite of possible selves is thus not the present or actual self but, as Heidegger reminds us, nothingness or the absence of possibility and, especially, of the possibility of becoming.

## Exploring Possible Pasts

While possible worlds, societies and selves are more or less easy to imagine, possible pasts are perhaps a harder concept to grasp – after all, the past is supposed to be singular and somewhat ‘objective’ given that it cannot be changed anymore. And yet, our relation to the past, either personal or collective, is just as complex and dynamic as the one with the future (discussed in the next section). This is because we never simply remember the past ‘as is’ (Bartlett, 1932), but constantly reconstruct it and wonder what might have happened if… These kinds of memory, thinking and imagination applied to the past open it to the possible and, in doing so, reinforce its connection with and relevance for the present and the future.

One of the clearest expressions of engaging with possible pasts is represented by counterfactual thinking. Counterfactuals are generally defined as conditional statements in which the first clause proposes something contrary to facts or to what is the case. While they can be applied to the future as well, most work in this area has been done on “alternative versions of past or present outcomes” (Roese & Olson, 1995, p. 1). Reflecting on what the present would be like if certain things in the past would have (or would not have) happened, is both a common exercise and one with powerful consequences. It not only helps us understand something about the past, present, and their relation, but it also can influence how we feel about them (could we have done anything to prevent a bad thing from happening? Or, conversely, helped something better happen?). In the literature it is common to distinguish between upward counterfactuals (when alternatives are better than actuality) and downward counterfactuals (when alternatives depict a worse state of affairs) (Roese, 1997). A lot of research over the past decades went into understanding how useful it is to employ each one of them. This led to functionalist models of counterfactual thinking, models that point to its value; for instance, downward counterfactuals can provide comfort while upward counterfactuals prepare the person for the future (see Markman, Gavanski, Sherman, & McMullen, 1993). In this sense, counterfactual thinking helps the individual engage not only with what might have been possible (the past), but also what is and could be possible (the present and future), effectively bridging these temporalities. One of the main limitations of old and new research into counterfactual thinking, however, is the overly cognitive description of this phenomenon as constructing and reconstructing mental models (see Byrne, 2017). Even when emotions are taken into account – and a lot of this research does study emotions – they are not considered outside an information processing framework.

A sociocultural approach to possible pasts is more readily employed in studies of autobiographical memory (e.g., de Saint-Laurent & Zittoun, 2018) and collective memory (e.g., de Saint-Laurent, 2018). In this kind of research, the person is considered in a social and developmental manner, as an agent that uses cultural means – such as symbols, narratives, life philosophies, etc. – to make sense of the past, present, and future, both individual and collective. Imagining collective pasts is not reduced here to singular mental processes, for instance counterfactual thinking, but seen as an activity in its own right, integrated within the life course and contributing to it. Within this framework, the link between collective memories and collective futures is emphasized, as well as that between possible selves and possible worlds. Indeed, giving new meanings to the past, and thus opening it to multiple possible interpretations, is fundamental for how people understand their place and their role in relations to others, in a shared society. The past is open to the possible precisely because it is not simply ‘there’, to be made sense of in an objective manner, by detached observers. This observation is not contesting in any way the existence of facts and the fact that some stories about the past are more accurate than others. The idea that there is always a sense of openness and possibility about the past should not be used to ‘rewrite history’ (as it sometimes is, unfortunately), but to understand it better by inviting multiple perspectives on it. In this way, the past can be reflected upon instead of taken for granted, and it can also serve as a reference point for building multiple visions of the future.

## Exploring Possible Futures

Most of the times we think about the possible what we actually consider are possible futures. This is not only because the future is inherently open (despite our best efforts to predict it as best we can), but also because we live lives that are fundamentally oriented towards the future (Valsiner, 2007). When reflecting on the latter, imagination is a key psychological process that comes to mind. And yet, in the last section I argued that memory, individual and collective, is shaped by (oftentimes implicit) visions of the future. Anticipations of the future or causal links made between past and future form the basis of counterfactual thinking and play an important part in making meaning about the past. At the same time, basic psychological processes such as perception, which are supposed to anchor us strongly in the here and now of the present, are equally future oriented. Gibson (1986), for instance, offered a classic account of visual perception that connects it to action and orients it towards discovering and enacting the affordances (or action possibilities) of the environments we live in. There is, therefore, ample support for the claim that our psychology is driven equally by the actual and the possible, the past and the future, and that this interplay transforms the present and our possibilities of acting within it.

It is not surprising then that the future received considerable attention in the human and social sciences for many decades. The field of Futures Studies finds its origins in the post-World War II era and the desire to build better, more peaceful and prosperous societies. According to Poli (2017, p. 59), “the two main principles underlying Futures Study are that the future is open and unknowable – notwithstanding all our efforts to argue in favor of the contrary – and that there is always a multiplicity of futures”. One of the most important and common distinctions in Futures Studies is that between forecasting and foresight. The former, widely found in a series of disciplines, from economy to meteorology, is typically grounded in quantitative analyses and predictive models. It starts from the premise that the world, both physical-biological and social, is organized around some given principles that govern both the past and the present. As such, knowledge about the past becomes useful for anticipating the future with different degrees of certainty. Although not labelled as forecasting, this kind of logic is common also in psychology, for example in developmental studies, whenever linear models of development are used, i.e., every person is assumed to necessarily pass through a series of pre-ordered stages. In contrast, foresight is not pointing to one – the most statistically likely – future, but a multiplicity of possible futures. It is more often qualitative in its analyses and focused on discontinuities between past and future. The most recent development within this area is concerned with both forecast and foresight and with the way they guide personal and collective decisions and actions. As noted by Poli in his *Introduction to anticipation studies*:

“An anticipatory behavior is a behavior that ‘uses’ the future in its actual decision process. Anticipation as here understood includes two mandatory components: a forward-looking attitude, and the use of the former’s result for action. A weather forecast in itself is not anticipatory in the sense used by this book. Watching a weather forecast and as a consequence taking an umbrella before going to work is instead an anticipatory behavior” (Poli, 2017, pp. 1-2).

This is an important observation that reveals the deeply pragmatic orientation of anticipation studies as an emerging field of research. Instead of reducing anticipation to a mental process, we are invited to connect it back to action and, through this, to the intentionality of the person and her interactions with the environment. As Hoffman (2016) rightfully observes, the ‘birth defect’ of information processing models was to assume that all cognition starts from stimuli. In reality, “organisms and above all human beings typically do not respond on stimuli but they almost always act in order to create the stimulations or situations they are striving for” (p. 21). This means that anticipations of the future are not merely a second phase or an outcome of information processing, they are *part and parcel* of the way we perceive, think about and act in the world. Possible futures are not simply envisioned by the person based on information about the past and the present, they are driving the ways in which we acquire and process this kind of information. And, by becoming actualized, over time, they create new conditions that lead to new anticipations and open new possibilities for the person, the group, or society as a whole.

This kind of theorizing is fruitful for novel concepts such as speculative thinking. The idea of speculation, as proposed by Savransky, Wilkie, and Rosengarten (2017), goes against usual associations with finance, for instance, and an excessive reliance on forecasting. “By contrast, speculation is associated with a sensibility concerned with resisting a future that presents itself as probable or plausible, and to wager instead that, no matter how pervasive the impasse may be, it can never exhaust the unrealised potential of the present” (p. 8). It is an invitation to reflect on what might seem impossible, to take risks, and to explore the plurality of the present. It is, as the authors aptly define it, a “struggle against probabilities” (p. 7), one aimed at keeping the future open and, with it, also our decisions and actions in the present. The impossible or the highly unlikely are also exploited by utopias and dystopias. Located in a distant or unspecified future, these literary projects are well anchored in past and present and they tend to exacerbate one or more of their dimensions. While offering concrete and detailed description of what societies could be like in the future, it would be wrong to assume that they are closing up possibilities. On the contrary, “in each instance, the specific utopias produce consequences that force a questioning of the original vision and that shape both its development and how individuals experience it” (Gordin, Tilley, & Prakash, 2010, p. 4). Whether intentionally or not, utopian and dystopian visions alike are meant to present us with the consequences of actualizing a certain future and, consequently, to help us reflect on it and consider alternatives. In doing so, they cultivate the kind of anticipations and speculations that open up – rather than close – possible futures.

## Exploring Possible Theories

The possible as a field of inquiry, as suggested until now, has both a long past and a short history. In many ways, and due to its profoundly interdisciplinary nature, it finds its roots in the philosophy of Antiquity and the Renaissance. At the same time, calls to consider the possible, possibility, and the future have intensified over the last two decades in a variety of disciplines across the social and natural sciences, including the humanities and the arts (both of them highly invested into experimenting with the possible). From research practices to interventions, these calls are starting to bear fruit. What about theoretical developments? What theories of the possible – of engaging and cultivating it – are available today?

This is a difficult question to answer given that, oftentimes, a relevant theory or philosophical orientation might not directly focus on the possible but on connected issues (agency, freedom, emergence, and so on). At the same time, it is hard to offer comprehensive overviews of theories of the possible in such a brief editorial. My focus in this last section will thus be necessarily selective and cover mainly psychological theories. Psychology as a discipline has an important part to play in the multi- or trans-disciplinary science of the possible. This is due to the fact that it deals itself with a topic – the human psychic – that is, on the one hand, central for any discussion of possibility and, on the other, situated at the intersection between multiple systems, from biological to societal.

In psychology, there are a number of recent candidates for ‘possible’ theories of the possible. One of them is prospection theory, put forward by Seligman, Railton, Baumeister, and Sripada (2013). In line with the general ethos of studying the possible, the authors proposed an incipient science of prospection as a way of changing psychology’s focus from past to future. Indeed, most schools within psychology, from psychoanalysis and behaviorism to cognitive science, tend to place a lot of weight on the past and to emphasize the fact that it shapes the present. In contrast, the notion of prospection reverses this relationship and claims that we are guided by the future. Importantly though, Seligman and colleagues specify that it is not the future itself, but evaluative representations of possible future states. As prospective organisms, we “construct an evaluative landscape of possible acts and outcomes” (Seligman et al., 2013, p. 120) and constantly revisit these representations. In summary, the cognitive model proposed includes expectations, observing reality, noting discrepancies, reducing them by changing the initial expectations, and repeating this cycle. The focus, predictable for cognitive psychology, is on information processing and typical biases in how we envision the possible. Is this the only way to theorize its dynamic?

Certainly not. A great counter-example is offered by the notion of possibility thinking developed over the last two decades by Craft and her collaborators (see Craft, 2015). Coined to capture the creative and developmental quality of interacting with others and the world across the life-span, possibility thinking revolves around common interrogations such as ‘what if’ and ‘as if’. In Craft’s (2015, p. 153) words:

“By asking ‘what if?’ the transition is made from ‘what is’ to ‘what might be’, or from ‘what is this?’ to ‘what can I/we do with this?’. (…) Such what if? thinking can be seen in the toddler who makes mud and grass ‘soup’, in teenagers designing hypotheses and fair tests in science and in adults taking a short cut in traffic or considering whether to introduce one friend to another”.

Not only is possibility thinking wider than the notion of prospection but, despite its use of the term ‘thinking’, it is anything but reducing the possible to mental processes. In fact, it considers it a highly embodied process of experiencing the world, one characterized by posing questions, playing, being immersion and imaginative, acting with self-determination and taking risks, among others. Possibility thinking is also, by definition, a social phenomenon as it can only be scaffolded by social interactions and the use of culture. These premises are common for sociocultural theories of mind, creativity, imagination, education, and society. While sociocultural psychology is a diverse and emerging field (more accurately referred to as sociocultural or cultural psychologies; see Valsiner & Rosa, 2007), its many branches are unified by an embodied, symbolic, social, and developmental conception of the mind. This level of complexity is particularly important for understanding the possible and, indeed, the possible as a notion has been – either implicitly or explicitly – central for most sociocultural authors (e.g., Vygotyky, Mead, Dewey, & Bakhtin). Particularly in recent years, sociocultural psychologists initiated substantial explorations of agency (Gruber, Clark, Klempe, & Valsiner, 2015), imagination (Zittoun & Gillespie, 2016) and creativity (Glăveanu, 2014), each one of them bringing new insights into the possible and its cultivation.

In this last segment of the editorial I will briefly sketch a new, sociocultural account of the possible, one that builds on what I formulated in the past as the perspectival model of creativity (see Glăveanu, 2015). The core argument behind the model is as follows: experiencing difference, in particular self-other differences, offers individuals and groups new perspectives from which to understand and act on the world. Creativity emerges out of putting these perspectives in dialogue with each other in a playful and reflective manner. It is by being able to ‘see’ a problem, situation or issue from multiple perspectives that we become freer, more flexible and more open-minded in relation to it. This increases our changes of being creative (although the mere presence of differences and even of different perspectives doesn’t guarantee, by itself, a creative outcome). At the heart of this argument is the claim that *what creative actions do is open up, exploit and expand the possible for both self and others*. In other words, *multiple perspectives, growing out of difference and enhanced by dialogue, are the basis of the possible* in all its forms of expression (possible worlds, possible selves, possible pasts and futures, among others). To experience the possible, one needs to become aware of differences in perspective and of the fact that we all live, as human beings, in a perspectival world. How do we gain this awareness to begin with? It is because we occupy various positions in this world – in physical, social, and symbolic terms – and that we interact with others from their position, that we diversify our range of perspectives in the first place. Being able to move between positions and adopt more than one perspective on the problem or issue at hand is a tremendous developmental achievement, a way of experiencing the world simultaneously as is and as different from how it is. The ontological history of engaging with the possible begins thus with differences of position and perspective and finds its expression in our capacity to occupy *meta-positions* vis a vis reality. The latter are fundamental for the experience of wondering (Glăveanu, 2017), an ever-present companion of exploring the possible.

The theory briefly outlined above has a series of conceptual and practical consequences. To start with, it proposes that the real or the actual are not the opposite of the possible. Since the possible is rooted in difference and the multiplicity of perspectives, its antithesis is *sameness* or the *absence (or denial) of difference*. We often encounter in our daily lives perspectives that are dominant, hegemonic, and overpowering – views that ‘hide’ their ontological existence as one perspective among many and, based on the rhetoric of truth, objectivity or sanctity, impose themselves as legitimate and singular. From science to religion and through nationalist and totalitarian discourses, individuals and groups are often subjected to monological, possibility-closing discourses and practices and fortunately, just as often they struggle to resist them. It is particularly painful to notice that such processes, that are averse to the possible and possibility, are often inscribed within education. This observation led generations of educators, psychologists and philosophers to question schooling and challenge its practices. Bruner (2007), famously spoke in one of his last lectures about “the crucial importance of cultivating a lively sense of the possible in the rising generation”; he continued: “knowledge (…) is not just what we store inertly in our heads. It also provides a launching pad into the realm of the possible. And cultivating the uses of knowledge as such a launching pad must (…) become a crucial task of the educational establishment” (Bruner, 2007, pp. 1-2).

A sociocultural theory of the possible is, indeed, incomplete without a *pedagogy of the possible*. Both of them find their place within the expanding, and extremely exciting, field of research and inquiry that has been introduced here. If multiple perspectives open up the possible, then the most important developments in this new/old field of study will come from systematically placing the conceptions above in dialogue, in particular dialogues that cut across disciplinary, cultural, geographic and temporal boundaries. The present editorial represents an open invitation to contribute new viewpoints to these exciting conversations.
